# Protein–Ligand Docking in the Machine-Learning Era

**DOI:** 10.3390/molecules27144568

**Published:** 2022-07-18

**Authors:** Chao Yang, Eric Anthony Chen, Yingkai Zhang

**Affiliations:** 1 Department of Chemistry, New York University, New York, NY 10003, USA; cy1063@nyu.edu (C.Y.); eac709@nyu.edu (E.A.C.); 2 NYU-ECNU Center for Computational Chemistry at NYU Shanghai, Shanghai 200062, China

**Keywords:** molecular docking, virtual screening, protein–ligand scoring function, machine learning, deep learning, datasets

## Abstract

Molecular docking plays a significant role in early-stage drug discovery, from structure-based virtual screening (VS) to hit-to-lead optimization, and its capability and predictive power is critically dependent on the protein–ligand scoring function. In this review, we give a broad overview of recent scoring function development, as well as the docking-based applications in drug discovery. We outline the strategies and resources available for structure-based VS and discuss the assessment and development of classical and machine learning protein–ligand scoring functions. In particular, we highlight the recent progress of machine learning scoring function ranging from descriptor-based models to deep learning approaches. We also discuss the general workflow and docking protocols of structure-based VS, such as structure preparation, binding site detection, docking strategies, and post-docking filter/re-scoring, as well as a case study on the large-scale docking-based VS test on the LIT-PCBA data set.

## 1. Introduction

Discovering bioactive compounds for a given target from a large compound library is one of the major tasks in drug development. It is laborious and costly to carry out binding affinity measurements on tens or hundreds of thousands of compounds. Hence, the overall cost of drug discovery will be greatly reduced if the binding affinities of compounds can be effectively predicted with computational methods before performing experiments. Employing computational methods to find active compounds is called Computer-Aided Drug Design (CADD) [1,2,3,4,5,6]. CADD has emerged as a powerful and promising technique in the development of new hit compounds and led to the discovery of several approved drugs, including the human immunodeficiency virus I (HIV-1) drugs (amprenavir and saquinavir), the fibrinogen antagonist (tirofiban), the carbonic anhydrase II inhibitor (dorzolamide), the angiotensin-converting enzyme (ACE) inhibitor (captopril) and the human rhinovirus 3C protease inhibitor (rupintrivir) [5,7,8,9,10].

CADD methods can be categorized into two general types, structure-based drug discovery (SBDD) and ligand-based drug discovery (LBDD) [11]. SBDD aims to find active compounds based on the physical interactions of 3-dimentional (3D) structures between the target protein and small molecule [2]. LBDD investigates existing activities using approaches of quantitative structure-activity relationship (QSAR) models, chemical similarity, pharmacophore and 3D shape matching to predict the property of a novel compound [12]. Both SBDD and LBDD are widely used in drug discovery processes and can be combined to use in virtual screening (VS). For example, scientists can first search for compounds similar to available and moderately active compounds from a large library using LBDD and then predict the protein–ligand interactions to find the favorable compounds using SBDD [8].

Understanding the binding mechanism of the protein and small molecule is crucial to discover and optimize drug molecules. The application of SBDD tools has gained significant interest in recent decades due to the recent explosion of high quality 3D macromolecule structures [2]. SBDD aims to identify binding sites and interactions that are important for the biological function of the protein. This structural information then suggests the design of therapeutic compounds that can compete with essential interactions involving the target protein and thus interrupt the anormal biological pathways.

Structure-based inhibitor design approaches often use molecular docking, a computational procedure that efficiently predicts non-covalent interactions between macromolecules (receptor) and small molecules (ligand) [13,14,15,16,17]. This procedure mimics the lock-and-key model of drug action to predict the experimental binding pose and affinity of a small molecule within the binding site of the target protein [18]. Docking methods are commonly used in structure-based VS on large molecular libraries, since they are fast enough to scan over millions of compounds using a simplified scoring function [19]. Docking programs, such as DOCK, AutoDock, GOLD, Glide, FRED and Surflex-Dock, rely on scoring functions to evaluate protein–ligand binding [15,17,20,21,22,23,24]. Therefore, the critical component of molecular docking is a robust, fast and accurate scoring function.

In this review, we will first describe protein–ligand scoring functions including its classification, datasets, and evaluation metrics. Then, we discuss recent advances in ML-based scoring functions. Finally, molecular docking protocols and general workflow utilized in structure-based VS are examined.

## 2. Protein–Ligand Scoring Functions

The binding affinity between a protein and ligand is determined by their binding free energy. Rigorous prediction of binding free energy requires extensive sampling of complex conformations and explicit treatment of aqueous solution environment, such as free energy perturbation (FEP) [25,26] and thermodynamic integration (TI) [27,28], which are too computationally expensive to be suited for large scale VS. Alternatively, molecular docking typically employs a scoring function to estimate the protein–ligand binding free energy based on a single protein–ligand complex structure. This is much faster and facilitates its use in VS on large molecular libraries [3,19,29].

**Figure 1:**
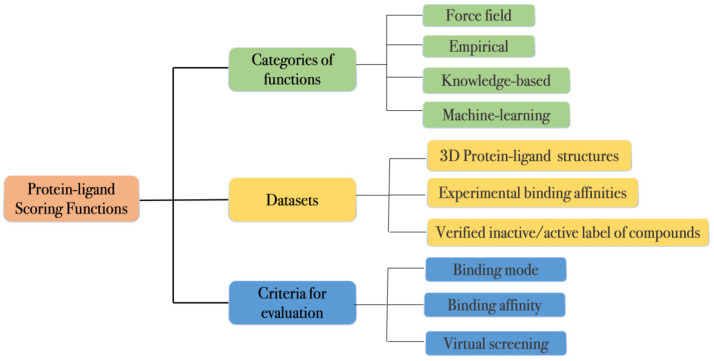
Schematics of the categories and datasets and evaluations of the protein–ligand scoring functions.

Scoring functions are a family of computational methods that have been widely applied in SBDD for fast evaluation of protein–ligand interactions [30,31]. They can be used in a molecular docking job to rank different putative ligand binding poses to select the most favorable one (best-scored pose). The score of the favorable pose is used to represent the binding affinity of the compound. This combined docking/scoring scheme has been widely applied to VS for hit identification as well as structure-activity relationship (SAR) analysis for hit-to-lead and lead optimization [6,29,32]. In this section, we will introduce protein–ligand scoring functions including its classification, datasets, and evaluation (as shown in Figure 1).

### 2.1. Classification

Scoring functions first emerged in the early 1990s and have inspired continuing research since then. Researchers have developed a variety of scoring functions formulated on different assumptions or algorithms [30,33,34]. These scoring functions can be roughly classified into four categories: (i) physics-based methods, (ii) knowledge-based statistical potentials, (iii) empirical scoring functions, and (iv) machine-learning scoring functions [35].

(1) Physics-based scoring functions are centered on molecular mechanical calculations [20,21,36]. These scoring functions are often predicated on fundamental molecular physics terms such as Van der Waals interactions (Lennard-Jones potential), electrostatic interactions (coulombic potential) and desolvation energies. These terms can be derived from both experimental data and ab initio quantum mechanical calculations. Due to the computational cost, solvation and entropy terms are usually oversimplified or ignored in physics-based scoring functions. Programs such as GoldScore, DOCK and early versions of AutoDock use this type of scoring function [20,21,36].
(2) Knowledge-based scoring functions consist of statistical potentials derived from experimentally determined protein–ligand structures. The frequency of specific interactions from many protein–ligand complexes are used to generate these potentials via the inverse Boltzmann distribution. This approach approximates complicated and difficult-to-characterize physical interactions using large numbers of the protein–ligand atom-pairwise terms. As a result, the scoring function lacks an immediate physical interpretation. DrugScore, ITScore and PMF are examples of knowledge-based scoring function [37,38,39,40].
(3) Empirical scoring functions characterize the binding affinity of protein–ligand complexes based on a set of weighted scoring terms. These scoring terms may include descriptors for VDW, electrostatics, hydrogen bond, hydrophobic, desolvation, entropy, etc. The corresponding weights of the descriptors are determined by fitting experimental binding affinity data of protein–ligand complexes via linear regression. Empirical scoring functions draw from both physics-based and knowledge-based scoring functions. Empirical scoring functions use physically meaningful terms similarly to physics-based scoring functions. The contribution (weight) of each term is learned from the training data, similarly to knowledge-based scoring functions. Compared to knowledge-based scoring functions, empirical scoring functions are less prone to overfitting due to the constraints imposed by the physical terms. The scoring terms also provide insight into the individual contributions to the final binding affinity. Bohm pioneered the first empirical scoring function, LUDI, in 1994 [41,42]. Other famous empirical scoring functions, such as ChemScore, GlideScore, X-Score and Autodock Vina, were developed afterwards [21,22,23,43,44]. Autodock Vina is one of the widely used open-source docking programs, and its scoring function consists of five empirical interaction terms (two gaussian terms, a repulsion term, a hydrogen bond term, and a hydrophobic term) and a ligand torsion count term [43]. Recently, a linear empirical scoring function inspired from Vina scoring function, Lin_F9, was developed to improve the scoring performance and overcome some of the limitations of Vina by introducing new empirical terms, such as the mid-range interactions and metal–ligand interactions. Trained on a small but high-quality protein–ligand dataset, Lin_F9 achieved better scoring accuracy than Vina in binding affinity prediction [45].
(4) Machine learning (ML) scoring functions are a group of methods that use ML techniques to learn the functional form of the binding affinity by associating patterns in the training data. Without employing a predetermined functional form, ML scoring functions can implicitly capture intermolecular interactions that are hard to model explicitly. ML scoring functions have shown marked improvements in binding affinity prediction in recent years [46,47]. In Section 3, we will discuss ML scoring functions in detail.

The first three types (1–3) can be grouped as classical scoring functions. These scoring functions usually adopt a linear form, which is a linear combination of several force-field or interaction descriptors. On the other hand, ML scoring functions can adapt much more complicated functional forms by utilizing ML methods, such as Support Vector Machine (SVM) [48], Random Forest (RF) [49], eXtreme Gradient Boosting (XGB) [50], Deep Neural Network (DNN), Convolutional Neural Network (CNN) and Graph Neural Network (GNN) [51,52].

### 2.2. Datasets

A representative dataset is the most substantial part of protein–ligand scoring function development and is crucial for the evaluation of scoring functions. Here, we introduce some widely used datasets:

(1) Datasets that consist of 3D protein–ligand structures with experimentally measured binding affinities are typically used to evaluate methods in binding pose identification and binding affinity prediction [53,54,55]. One example, PDBbind, provides 3D protein–ligand structures with experimentally measured binding affinity data manually collected from their original references. PDBbind is currently one of the largest datasets of protein–ligand structures for the development and validation of docking methodologies and scoring functions. The current release (version 2020) of PDBbind general set contains 19,443 protein–ligand complexes with binding affinity data (K_d_, K_i_ or IC_50_) ranging from 1.2 pM to 10 mM, and is annually updated to keep up with the growth of Protein Data Bank (PDB) [56,57]. PDBbind also contains a refined subset of high-quality data according to several criteria concerning the quality of the structures and the affinity data. In addition, PDBbind provides a benchmarking “core set” used for the comparative assessment of scoring functions (CASF) [33,34], which will be discussed in Section 2.3 in detail. Similar datasets, such as the Community Structure-Activity Resource (CSAR) [58,59,60,61,62,63] exercises and the D3R Grand Challenge [64,65,66,67], are mainly curated to validate SBDD.
(2) Datasets that label active/inactive compounds to protein structure or sequence targets are generally used to develop and evaluate methods in VS tasks, such as early hit enrichment and active/inactive classification [68]. The Database of Useful Decoys (DUD) [69] and Database of Useful Decoys-Enhanced (DUD-E) [70] have been widely used for benchmarking. DUD-E consists of 102 targets with 22,886 active compounds with binding affinities. For each active compound, DUD-E also includes 50 computer-generated decoy compounds, which have similar physiochemical properties but dissimilar two-dimensional topology to the active compound. Many decoys are presumed, without experimental verification, to be inactive compounds. This remains a major drawback for DUD-E dataset because false negative samples might exist in the dataset.

Maximum Unbiased Validation (MUV) database is constructed based on PubChem bioactivity data from 17 targets, each with 30 actives and 15,000 inactives, and is designed to avoid analog bias and artificial enrichments [71]. Unlike DUD and DUD-E, MUV provides experimentally verified inactive compounds mostly tested with cell-based assays. This raises questions into the suitability of using MUV as a structure-based VS benchmark because many actives are not validated against their putative targets. Thus, MUV is more appropriate for benchmarking ligand-based VS approaches.

In 2020, Tran-Nguyen and co-workers have curated LIT-PCBA [68], a dataset derived from dose–response assays in the PubChem database [72,73,74]. LIT-PCBA consists of 15 targets, and for each target, all the actives and inactives were taken from the experimental data under homogeneous conditions. One main advantage of LIT-PCBA over prior efforts is the careful removal of potential false-positive results (dose–response curve of each active should have 0.5 < Hill slope < 2.0). However, the main limitation of LIT-PCBA dataset is that more than half of the primary assays (8 of 15 targets) are cell-based phenotypic assays. Thus, structure-based VS tests on this benchmark also have some limitations.

(3) Other datasets contain a large variety of compounds with binding affinity data but contain few or lack annotated protein–ligand structures, such as the Binding Database (BindingDB) and ChEMBL. These are used in developing ligand-based or sequence-based approaches to predict binding affinities and can supplement protein–ligand scoring function development and validation [75,76,77,78,79,80,81,82]. As of 4 May 2022, BindingDB contains 2,513,948 binding data for 8839 protein targets and 1,077,922 small molecules. ChEMBL is a manually curated database of bioactive molecules with drug-like properties. The current release (version 30) contains 19,286,751 activities for 14,885 targets and 2,157,379 compounds.

### 2.3. Evaluation Metrics

Several metrics are commonly used to evaluate the performance of a scoring function in binding pose identification, binding affinity prediction, and VS tasks.

(1) The goal of binding pose identification is to determine the native binding pose among computer-generated decoys. Given a set of decoys, a reliable scoring function should be able to rank the native binding pose to the top by their binding scores. The root-mean-square deviation (RMSD) between the top docking pose and the experimentally determined ligand pose is a commonly used evaluation metric. If the RMSD is ≤2 Å, the binding pose prediction is considered successful. Due to its simplicity and ease of implementation, the RMSD metric for binding pose prediction has been widely used in the field [33,34,64,65,66,67,83]. It should be noted that the minimum symmetry-corrected RMSD should be calculated for small molecules with symmetric functional groups or whole-molecule symmetry [84,85,86,87].
(2) Binding affinity prediction aims to predict the binding affinity for a given protein–ligand complex. Nevertheless, some scoring functions give a score that cannot be directly compared to experimental binding data [20,88]. Thus, a widely used criterion for affinity prediction is the Pearson correlation coefficient between the predicted scores and the experimental binding data on benchmark test sets [33,34]. Since the correlation between the predicted scores and experimental binding data does not have to be linear, an alternative criterion is the Spearman ranking correlation coefficient. This first ranks the predicted and experimental scores and then calculates the correlation between the two ranking sets [89].
(3) VS aims to identify true actives in a compound library. The screening performance typically estimates whether a scoring function is able to rank the known binders above many inactive compounds in the library. There are several evaluation metrics, including enrichment factor (EF), area under the curve (AUC), and receiver operating characteristics (ROC) curve, to quantify the screening performance of a scoring function [90]. EF is defined as the accumulated rate of true binders found above a certain percentile of the ranked database that includes both the actives and inactives. A higher EF at a fixed percentage of ranked database indicates better early hit enrichment (a higher likelihood to select actives based on predicted scores). EF is computed as follows:
  (1) $$EF_{\alpha }=\frac{NTB_{\alpha }}{NTB_{total}\cdot \alpha },$$
  where $NTB_{\alpha }$ is the number of true binders among top α percentile of ranked candidates (e.g., α = 1%, 5%, 10%) based on predicted binding scores and $NTB_{total}$ is the total number of true binders in the database. AUC-ROC is an evaluation method for classifiers assessing true binder identification. ROC plots the false positive rates (FPR, also called specificity) and true positive rates (TPR, also called recall or sensitivity) into a curve whose area under the curve (AUC) ranges from 0 to 1, where 0.5 reflects a random-level selection and 1 for perfect selection. This method is more appropriate when the number of inactive compounds is comparable to the number of active compounds.

The comparative assessment of scoring functions (CASF) benchmark is one of the most widely used retrospective benchmark for evaluation of scoring functions [33,34]. The current version (CASF-2016) is derived from PDBbind refined dataset, consisting of 57 targets with 5 crystal protein–ligand complexes for each target (5 different co-crystallized ligands from high affinity to low affinity in order) [54]. Scoring functions are evaluated on four different metrics (scoring, ranking, docking, and screening). The scoring metric calculates the Pearson correlation coefficient between predicted binding scores and experimentally measured binding affinities. The ranking metric measures the average Spearman’s rank correlation coefficient after ranking all 57 targets by their predicted binding score. This quantifies the ability of a scoring function to correctly rank the known ligands of a certain target protein. The docking metric calculates the rate which predicted poses are found to be similar to the crystal pose (RMSD ≤ 2 Å). This establishes the ability of a scoring function to find the native pose among computer-generated decoys. The screening metric incorporates two indicators to measure the ability of a scoring function to find true binders within the top 1%, 5% and 10% ranked ligands for each target. The first indicator is the success rate in identifying the average highest-affinity binder over all the targets and the second indicator is the average EF over all the targets. The screening power assessment of CASF benchmark is limited due to the small size of the database (only 285 compounds in total) and the lack of verification of inactive compounds for targets. Therefore, a large dataset with many confirmed inactive compounds, such as LIT-PCBA benchmark [68], should be more suitable to evaluate the early hit enrichment performance of a scoring function.

Annually over the years 2015–2019, the Drug Design Data Resource (D3R) has provided blind and open competitions for pose prediction and affinity ranking to evaluate participants’ computational methods [64,65,66,67]. In the last round (D3R Grand Challenge 4) [67], D3R has organized a multiple-stage competition for pose prediction (stage 1a and stage 1b) and affinity ranking (stage 2) for macrocyclic small molecule inhibitors targeting beta secretase 1 (BACE1), as well as a one-stage affinity ranking competition for Cathepsin S (CatS) inhibitors. Mean/median RMSD values are used to evaluate the participant’s prediction performance. Spearman and Kendall ranking correlation coefficients are used to assess the affinity prediction.

In early 2022, Critical Assessment of Computational Hit-finding Experiments (CACHE) [91], a prospective benchmarking project to evaluate and improve VS methods in real screening, has been launched and is open to public. The CACHE aims to organize multiple rounds of challenges to provide opportunities for scientists to improve and test their VS methods in next several years. These prospective benchmarks would enable the improvement of computational methods to handle novel targets in the future.

## 3. Machine-Learning Scoring Function

ML is a branch of artificial intelligence that has gained attention in diverse research fields, including CADD. With the rapid progress of computational power and exponential increase of data, ML has been applied in many branches of CADD, such as chemical space exploration, molecular property prediction, protein structure prediction and VS [92,93,94,95,96,97]. ML algorithms have also been widely employed in SBDD, such as pose prediction, binder/nonbinder identification and binding affinity prediction [46,96]. This review will focus on the discussion of ML scoring functions, a supervised learning method that learns from structure data labeled with experimental measured binding affinities. Early efforts used traditional ML methods, such as SVM, RF and GBT, to improve scoring performance on benchmark test sets. Their inputs were manually designed descriptors, such as molecular interaction fingerprints, ligand features, atom-pairwise terms and force field terms [46]. To date, many DL scoring functions have also been developed. However, they do not always significantly outperform traditional ML scoring functions [98]. In the following part, we will describe some ML scoring functions, as shown in Table 1.

RF-score, the first ML scoring function to outperform classical scoring functions on scoring tasks, was proposed by Ballester and Mitchell in 2010 [99]. It utilized the random forest algorithm with feature selection comprised of protein–ligand atom-wise pair counts at a predefined distance cutoff. In 2013, Zilian and co-workers proposed SFCscore^RF^ [100], which also utilized the random forest algorithm, but with feature selection of 63 empirical terms comprised of ligand-dependent descriptors (such as number of rotatable bonds), specific interactions descriptors (such as hydrogen bonds and aromatic interactions) and surface characteristics (such as polar and hydrophobic contact surfaces). The scoring performance of SFCscore^RF^ on two common benchmarks (CASF 2013 and CSAR-NRC HiQ) also significantly outperformed classical scoring functions. However, both RF-score and SFCscore^RF^ performed much worse on docking and screening tasks compared to classical scoring functions [116,117].

To address this issue, in 2017, the Zhang group employed a Δ-machine learning approach, in which a ML model was employed to parametrize corrections to the Vina score [101]. This strategy enabled the scoring function to have both the excellent docking power of Vina and the accurate scoring performance of the ML method. In their work, Δ_vina_RF_20_ was developed using random forest with feature selection comprised of 10 features related to pharmacophore-based solvent-accessible surface area (SASA) and 10 empirical terms from Vina 58 features. As a result, Δ_Vina_RF_20_ achieved the best performance among a panel of classical scoring functions in all evaluation metrics (scoring, ranking, docking and screening powers) for the CASF 2007 and 2013 benchmarks. In 2019, the same group proposed a subsequent scoring function, Δ_Vina_XGB [102], that considered explicit water molecules as well as ligand conformation stability and substituted the random forest method with eXtreme Gradient Boosting (XGB). The feature set of Δ_Vina_XGB consisted of Vina 58 features, 30 SASA features, 3 bridge water features, 2 ligand stability features and 1 metal count term. The training data was enlarged to include both explicitly solvated protein–ligand structures and dry protein–ligand structures, as well as docking decoys. This Δ_Vina_XGB consistently achieved better performances in scoring, ranking, docking and screening tasks on the CASF-2016 benchmark [102]. In 2022, a newly developed delta ML scoring function, Δ_LinF9_XGB [103], used a series of gaussian terms to characterize protein–ligand interactions in different distance ranges and further enlarged the training set to include more weak binders and docking poses. Δ_LinF9_XGB achieved superior scoring, ranking and screening performances on the CASF-2016 benchmark. In addition, Nguyen and Wei proposed an algebraic graph theory-based scoring function, AGL-Score [105], which achieved superior scoring, ranking, docking and screening performance on the CASF-2013 benchmark. This method was a gradient boosting trees (GBT) model integrating weighted algebraic subgraph features of protein–ligand complexes.

Recently, customized protein–ligand interaction features became popular in scoring function development, such as ET-score (2021) and ECIF-GBT (2021) [104,106]. ET-score employed protein–ligand interaction features defined by distance-weighted interatomic contacts between atom type pairs of the protein and ligand. ET-Score achieved very good scoring performance (with Pearson R = 0.827) on CASF-2016 benchmark. ECIF-GBT used extended connectivity interaction features (ECIF), which are a set of protein–ligand atom type pair counts that consider each atom’s connectivity to define the pairwise types. ECIF-GBT achieved Pearson R of 0.857 on CASF-2016 benchmark. However, both ET-score and ECIF-GBT were trained solely on crystal structures and their performances on docking and screening tasks remain an issue.

Besides the above introduced traditional ML scoring functions, DL models were also applied in protein–ligand scoring function development. Durrant and McCammon proposed two models using neural networks, NNScore 1.0 and NNScore 2.0 [107,108]. NNScore 1.0 employed a simple neural network composed of only one hidden layer with five neurons to classify the active and inactive compounds based on 194 features including both interaction and ligand-dependent terms. Comparatively, NNScore 2.0 included many more interaction terms and estimated the pK_d_ rather than an active/inactive classification. In 2017, Wallach and co-workers introduced AtomNet [109], the first CNN-based scoring function incorporating 3D structural information. The inputs of AtomNet used vectorized 3D grids placed over the protein–ligand interaction interface, with each grid cell storing a value describing the presence of some basic structural features, varying from a simple enumeration of atom types to more complex descriptors. Its network topology was made up of an input layer, followed by four 3D convolutional layers and two fully connected layers, and topped by a logistic-cost layer that assigns probabilities over the active and inactive classes. AtomNet achieved much better AUC value than Vina (classical scoring function) on the DUD-E test set. Several similar CNN-based scoring functions, such as the CNN model proposed by Ragoza et al. (2017) [118], Pafnucy proposed by Stepniewska-Dziubinska et al. (2017) [110], and Kdeep developed by Jimenez et al. (2018) [111], were published afterward. One of the limitations of these CNN models was the dependency on the coordinate frame. Different orientations of the same protein–ligand structure could generate different representations. In order to address this issue, Zheng and co-workers introduced OnionNet in 2019 [112]. This method used a CNN model with inputs based on rotation-free element-pair specific contacts between protein and ligand in different shells. OnionNet, as well as the subsequent OnionNet-2.0 (2021) [119], achieved excellent scoring performance on the CASF-2016 benchmark.

Deep graph neural network (GNN) methods also became popular in protein–ligand scoring function development. In 2018, Feinberg and co-workers introduced PotentialNet [113], which used a graph convolutional neural network (GCNN) to directly learn protein–ligand structures in terms of both intramolecular and intermolecular interactions. This approach consisted of three major steps to achieve feature learning, including covalent-only propagation, dual noncovalent and covalent propagation, and ligand-based graph gathering. The aggregation of updated ligand atomic vectors was used to predict binding affinity. PotentialNet achieved Pearson R of 0.822 on CASF-2007 benchmark. In 2019, Lim and co-workers proposed a GNN model with distance-aware attention mechanism to differentiate the contribution of each interaction to binding affinity [120]. Their GNN model was also designed to focus on intermolecular interactions rather than memorize certain patterns of ligand molecules. As a result, this GNN model achieved very good performance in terms of both VS and pose prediction. Recently, several novel GNN models, such as graphDelta (2020) [114], graphBAR (2021) and SIGN (2021) [115,121], were published, however, these GNN models did not outperform some of above-mentioned traditional ML scoring functions on the CASF benchmarks.

All the above discussed ML scoring functions were generic scoring functions which aim to perform well on all kinds of target proteins. However, this aim could be hard to achieve due to the particulars of each target. Recently, target-specific ML scoring functions were proposed to focus on a certain target [122,123,124]. These functions learned from training data of a certain target or family to deal with the special characteristics of the target. A target-specific approach could achieve state-of-the-art performance on well-studied targets with sufficient training data, but it would not be applicable for a novel target with little experimental data available.

## 4. Structure-Based Virtual Screening

VS is a computational approach used to identify chemical structures that are predicted to have particular properties. In drug discovery, it involves computationally searching large libraries of chemical structures to identify those structures that are most likely to bind to a target protein. Structure-based VS, also known as target-based VS, attempts to predict the best interaction of a ligand against a target protein to form a complex and employs scoring functions to estimate the binding affinity of the protein–ligand complex [125]. As a result, all the ligands are ranked according to their binding scores to the target, and the high scoring ligands are selected for experimental measurement. In recent decades, advances in VS have been made in the following:

(1) There have been developments in structure-based VS approaches, including improvements in sampling and scoring methods, that have resulted in significant improvements in docking, scoring and screening performances [46].
(2) Developments in GPU processing speeds and cloud computing have dramatically increased computational power. Researchers are now able to computationally process vast numbers of compounds in the drug-like chemical space.
(3) Advancements in structural biology (such as X-ray, NMR and cryo-EM) and computational protein structure prediction (such as AlphaFold2 and RoseTTAFold) [95,126,127,128] have allowed access to many more 3D structures.
(4) The number of compounds that are commercially available or can be readily synthesized has grown dramatically in recent years. For example, as of March 2021, the WuXi GalaXi and Enamine REAL Space collections contain 2.1 billion and 17 billion compounds, respectively [129]. In June 2022, the WuXi GalaXI and Enamine REAL Space collections have grown up to 4.4 billion and 22.7 billion compounds, respectively.

The convergence of these breakthroughs has positioned structure-based VS to be a promising direction for the discovery of novel small molecule medicine. With the appropriate computing infrastructure, it becomes practical to virtually screen ultra-large compound library (synthesized or purchasable) to find virtual hit compounds, some of which (usually up to 100 compounds) can be experimentally tested.

### 4.1. Molecular Docking Protocol

**Figure 2:**
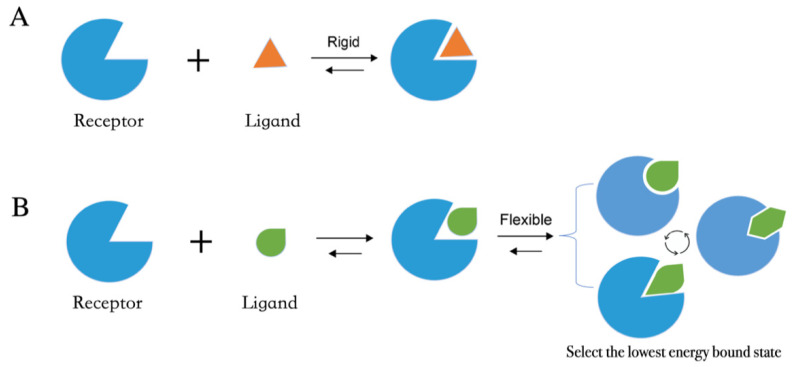
Two models of molecular docking. (**A**) A lock-and-key model. (**B**) Induced fit model.

Molecular docking methods predict receptor–ligand interactions at an atomic level and are widely utilized in structure-based VS. The docking process samples the optimal conformation based on the complementarity between the receptor and the ligand. Figure 2A shows the initially proposed “lock-and-key model”, which refers to the rigid docking of receptor and ligand to find the correct orientation for the “key” to open the “lock”. This model emphasizes the importance of geometric complementarity [18]. However, the real binding process is very flexible whereby the receptor and ligand changes their conformation to complement each other well. As shown in Figure 2B, the induced fit model considers structural flexibility and selects the lowest-energy bound state. Currently, major limitations of docking methods include a restricted sampling of both ligand and receptor conformations in pose prediction, as well as the previously discussed limited accuracy of scoring functions in affinity prediction.

The methods that improve sampling of ligand conformations can be defined as (i) incremental ligand construction, (ii) multiple conformers generation for docking and (iii) stochastic sampling [130]. In the first approach, the ligands are partitioned into small fragments that are individually docked into the receptor pocket according to the geometric fit. Docked fragments are then incrementally assembled to form an entire ligand within the binding pocket [131]. In the second approach, multiple low-energy conformations of the ligand are generated at first, and then individually docked against the receptor pocket [132].

The third and widely used strategy to account for ligand flexibility are stochastic methods, such as Monte Carlo (MC) or genetic algorithm (GA). MC algorithm, also known as simulated annealing, simulates docking by randomly generating minor changes in the position, orientation or conformation to generate new poses that are accepted or rejected based on the Metropolis acceptance algorithm [133]. The modeling begins at a high temperature such that there is a high probability of accepting the next conformation sampled. Then, the temperature is progressively decreased to reduce the conformational freedom of the system and to capture the receptor–ligand complex in a low energy state. GA employs a different approach inspired by Darwin’s theory of evolution [134]. The ligand begins as a random population of position, orientation and conformational states modeled as a set of chromosomes. Then, random crossovers and mutations are performed to produce another set of conformations. The conformation with the lowest binding energies with the receptor is accepted and then used to produce a new generation. This cycle is iteratively repeated until the local energy minimum of the receptor–ligand complex has been reached.

Many proteins possess varying degrees of flexibility, which can range from a slight perturbation of the ligand binding pocket to a complete reconstitution of the pocket. Therefore, an inadequate sampling of protein flexibility can result in an increase of both false positives and false negatives in VS experiments. Several approaches have been developed to tackle the issue of protein flexibility in recent years [135]. One common approach, named “ensemble docking”, is to utilize multiple receptor conformations in docking runs and to select the best-scoring conformation for further investigation [136,137,138]. The receptor conformations are commonly obtained from different X-ray and NMR structures or by sampling structures from molecular dynamics (MD) simulations. For instance, Abagyan and co-workers have investigated strategies for the selection of experimental protein conformations for VS and have found that the use of ensemble conformations of receptors co-crystallized with larger ligands provided the best results [139,140]. However, it has been noted that the use of excessively large numbers of receptor conformers in ensemble docking can lead to an increased number of false positive samples and linearly increased computational costs [135,141]. To alleviate some of these performance issues, ML techniques can be employed to help classify active and inactive compounds following ensemble docking [142]. Chandak and co-workers have tested multiple supervised ML methods trained on the DUD-E database to learn the relationship of a compound’s predicted binding affinities to the classification task.

An alternative approach to account for protein flexibility is to employ “soft docking”, where the interactions between the protein amino acid sidechains and the ligand is iteratively changed to allow partial clashing between the atoms of the protein and ligand [143]. For example, Ravindranath and co-workers have proposed a soft docking program, AutoDockFR [144], which simulates sidechain flexibility by sampling a large number of explicitly specified receptor sidechains and searching for energetically favorable binding poses for a given ligand. AutoDockFR optimizes protein–ligand interactions using the AutoDock4 force field and using a GA method combined with a Solis-Wets local search. This soft docking approach has achieved better binding pose prediction compared to rigid protein docking protocols but has also been associated with an increased number of false positive hits in structure-based VS [145].

### 4.2. Workflow in Virtual Screening

**Figure 3:**
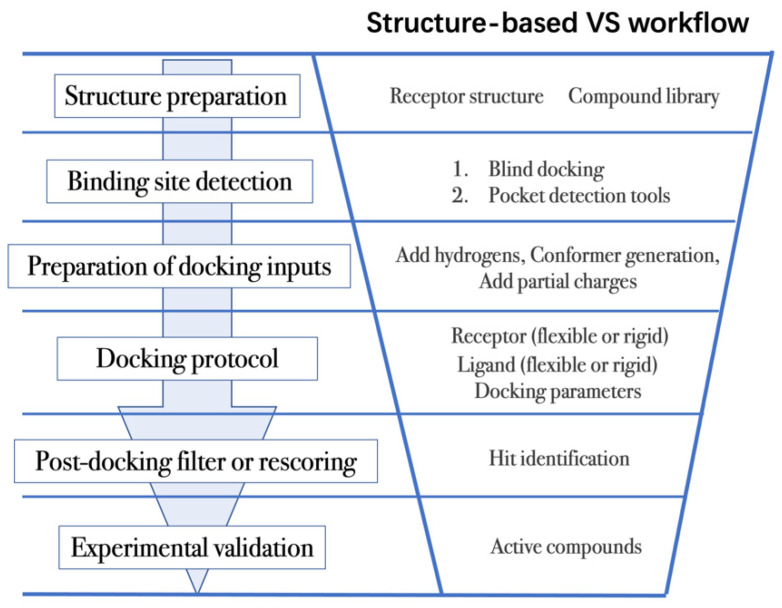
General scheme of a VS workflow.

Structure-based VS relies on docking of large collections of compounds into the binding pocket of target protein, and then evaluating whether the protein–ligand contacts will drive binding. As shown in Figure 3, the general VS workflow can be as follows:

(1) The first step is to obtain the 3D structures of a given target as well as the compound library. Experimental determined structures can be readily retrieved from the Protein Data Bank (PDB) [146], in which more than 120,000 unique protein structures have been determined through an enormous experimental effort. However, this represents a small fraction of the billions of known protein sequences whereby the 3D structure of a novel target is usually not available. In order to overcome this limitation, traditional computational prediction methods (such as homolog modelling and ab initio modelling) [147,148], as well as the recently developed DL methods (such as AlphaFold2 and RoseTTAFold) [95,127] can be employed to obtain the 3D structures of target proteins. In addition, the compound library or chemical space used in VS is also vital for hit identification.

As discussed above there is a growing number of options to dock to. It is important to note that the selection of which structure to dock to is not trivial. Docking results will differ depending on the conformation, apo/holo status, and quality of structure. One method, screening performance index, can be used to select good structures to use in prospective VS [149]. This index consists of five calculated terms that describe the docking performance of a set of structures on a set of known active compounds. Their testing has generally indicated that co-crystal structures with large ligands bound score well on the index and can be picked for prospective studies. These methods are limited because they require labeled datasets which may not be available for novel targets.

Compound libraries of approved drugs, natural products, already synthesized or purchasable compounds/fragments are commonly used in VS campaigns [29,130,150]. The well-known ZINC database contains over 750 million purchasable compounds, including over 230 million compounds in ready-to-dock 3D formats [151,152]. Recently, Jiankun and coworkers performed docking-based VS using a ultra-large compound library (more than 100 million compounds from ZINC make-on-demand compounds) to discover inhibitors targeting AmpC β-lactamase and D_4_ dopamine receptor [29]. Other databases, such as DrugBank [153,154,155] and Human Metabolome Database (HMDB) [156,157,158,159] are used to repurpose the approved drugs or human metabolites to the novel targets.

(2) The next step is to detect the binding site. Typically, the binding pocket on which to focus the docking calculations is known. For example, the binding site is chosen based on the information of co-crystallized ligand/substrate binding site, such as ATP binding site or protein–protein interactions (PPI) interface. However, when the binding site information is missing or a novel binding pocket needs to be explored, there are two commonly employed approaches, “blind docking” simulation [160,161] and pocket prediction algorithms. The first approach uses docking methods to search over the entire target structure to find a favorable ligand binding site, but it has a high computational cost in sampling. For the second approach, several available software can be employed to detect binding pockets, including AlphaSpace [162,163], FTMap [164], MDpocket [165], Fpocket [166], SiteMap [167] etc. These methods detect concave pockets on the protein surface by characterizing the spatial composition of amino acids or using the chemical probe to find favorable hot spots. Since drug resistance can arise for the orthosteric site of target proteins, these methods can be used to identify additional binding pockets that can be exploited for the design of novel inhibitors, such as allosteric or cryptic pockets [168,169].
(3) Once the binding site is determined it is important to carefully prepare docking input files to achieve successful VS. The preparation of protein structures starts from the assignment of protonation states for the amino acids, which can be done using software including PROPKA [170], H++ [171], and SPORES [172]. Then hydrogen atoms and partial charges are assigned. A popular software for this task is PDB2PQR [173,174]. In addition, the consideration of water molecules and metal ions can be crucial in certain target structures. Explicit water molecules mediating protein–ligand interactions should be analyzed and can be used to identify water-mediated interactions and avoid incorrect binding poses [175,176,177]. It is also important to consider coordination interactions between metal ions and ligand molecules for metalloprotein complexes [45,178].

Unlike proteins, most compounds used in VS are stored in line notation, such as Simplified Molecular Input Line Entry Specification (SMILES) string [179]. The 3D atomic coordinates of these compounds can be obtained from the line notation using several opensource softwares, such as RDKit and Openbabel [180,181,182], or commercial softwares, such as Omega and ConfGen [183,184,185]. Ligand protonation is also important since it affects the net charge of the molecule and the partial charges of individual atoms. Different docking programs will employ different charge assignment protocols. For example, AutoDock uses Gasteiger-Marsili atomic charges whereas the AutoDock Vina does not require the assignment of atomic charges, since the scoring terms that compose its scoring function are charge-independent [43,186].

(4) After the input files are created, the appropriate docking protocol must be selected. As has been discussed in Molecular Docking Protocol (Section 4.1), there are many different docking protocols that consider protein and ligand flexibility to enhance the performance of pose prediction. One of the most commonly used protocols is to perform flexible ligand–rigid receptor docking for each docking run, and then dock multiple protein conformations using the ensemble docking strategy [139]. In addition, several docking programs can be combined to avoid the limitations of one algorithm. For instance, Ren and co-workers have explored the effects of using multiple softwares in the pose generation step [187]. They use a RMSD-based criterion to come up with representative poses derived from 3 to 11 different docking programs. The resulting pose prediction achieves better performance than that of each individual docking program.
(5) Following docking, the results can be rescored or filtered. The computer-generated poses are evaluated based on the ability of the docking protocol to (i) select favorable binding poses for each ligand, and (ii) rank the ligand library to select high scoring hits for experimental measurement. Although the docking calculations are fast enough to process large compound libraries, they suffer from the inherent problem of calculating binding affinities from several simplified scoring terms. One remedy for improving the performance of VS is to employ more rigorous free energy calculations to postprocess docking poses. The main limiting factor in the application of free energy calculations to large chemical libraries is the high computational cost.

In recent years, post-docking filter methods have gained significant interest in drug discovery because they usually provide higher hit rates in VS with low additional computational cost and result in better correlation with experimental data in retrospective benchmarks. Several methods have been designed to eliminate false positive hits obtained from the initial docking experiments. Marcou and co-worker proposed the use of molecular interaction fingerprints (IFP), which are simple bit strings that convert the 3D information of protein–ligand interactions into a 1D vector representation, for the screening of CDK2 inhibitors [188]. The authors demonstrate that using post-docking filters that calculate the Tanimoto similarity of IFP between docked pose and co-crystal pose is more statistically accurate compared to classical scoring functions in discriminating active compounds from inactive ones. They base this on the assumption that active compounds should have certain specific interactions or contacts with their target to display activity. Bertho and co-workers reported a similar post-docking filtering strategy, namely automatically analyzing poses using self-organizing map (AuPoseSOM) to examine the interatomic contacts between the ligand and the target [189]. This type of approach is target-specific and requires the co-crystal ligand pose as the reference. ML can also be applied to this task. Stafford and co-workers introduced AtomNet PoseRanker, a graph CNN trained on PDBbind v2019 to rerank putative co-crystal poses [149].

Another post-docking strategy is the rescoring of docked poses using a consensus model or an advanced ML scoring function. On one hand, the consensus model uses several different scoring functions to re-assess the docking poses generated from a single docking algorithm. Charifson and co-workers have proposed an approach that takes the intersection of the top-scoring molecules according to two or three different scoring functions. They found it provides a dramatic reduction in the number of false positives identified by individual scoring functions on case studies of p38, IMPDH and HIV protease [190]. On the other hand, advanced ML scoring functions developed in recent years, such as AtomNet [109], vScreenML [191], Δ_Vina_RF_20_ [101], Δ_Vina_XGB [102], SIEVE-Score [192] and RF-Score-VS [117], outperform classical scoring functions in screening performance comparisons on benchmark test sets. However, there is no guarantee that ML scoring functions can outperform classical scoring functions on novel targets that are largely different from the samples in the training data set [193].

The above (1) to (5) steps summarize the workflow of VS process. Other structure-based approaches, such as MD simulations, have also been widely utilized in combination with docking to improve VS performance. MD simulations are an efficient approach to discover cryptic binding pockets (in step 2, binding site detection) [169,194], to sample multiple receptor conformations in ensemble docking (in step 4, docking protocols) [136], and to evaluate the interactions of the predicted receptor–ligand complexes (in step 5, post-docking analysis) [195,196].

### 4.3. Case Study

**Figure 4:**
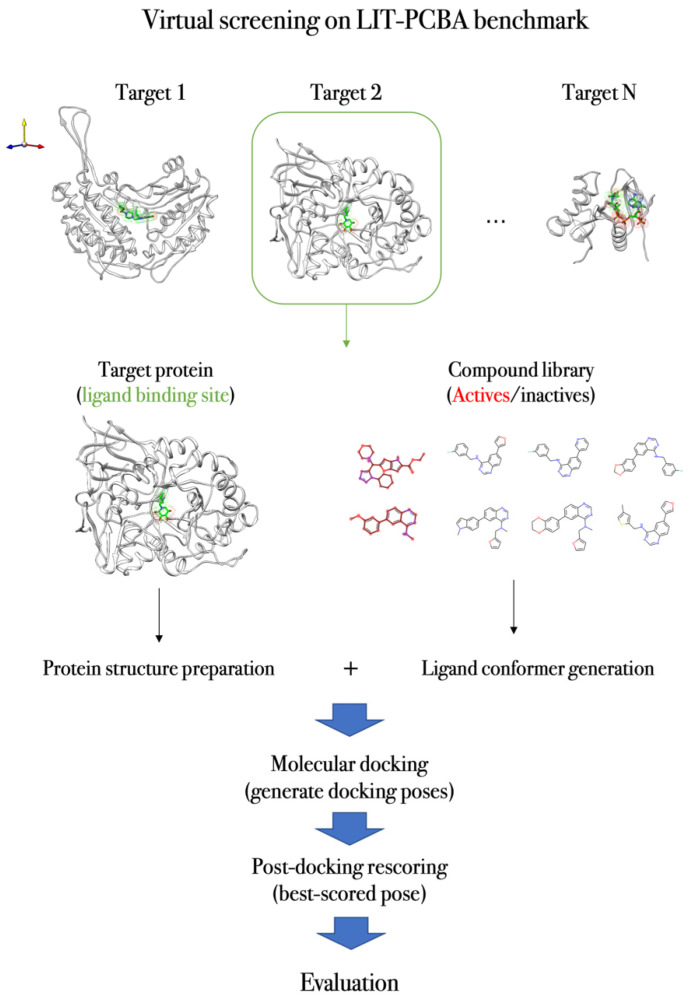
Workflow of docking-based VS protocol on LIT-PCBA benchmark.

To illustrate one virtual screening approach, we describe the application of Δ_Lin_F9_XGB VS protocol on the LIT-PCBA benchmark dataset [103]. The LIT-PCBA benchmark (discussed in more detail in Section 2.2), contains 15 diverse target proteins and the corresponding curated active/inactive compound library from the PubChem BioAssay database [68]. The target protein has one or several PDB structures, in which the co-crystal ligands are used to determine the docking box. The compound library contains SMILES strings of active and inactive compounds, which are processed with RDKit [181] to generate and protonate low energy 3D conformers for each ligand. Then, flexible ligand-rigid receptor docking is performed using the Smina program with Lin_F9 scoring function. After docking, the top 5 docking poses were re-scored using Δ_Lin_F9_XGB, and the best-rescored pose was used for VS assessment [103]. Figure 4 illustrates the general workflow of docking-based VS protocol on the LIT-PCBA benchmark.

**Figure 5:**
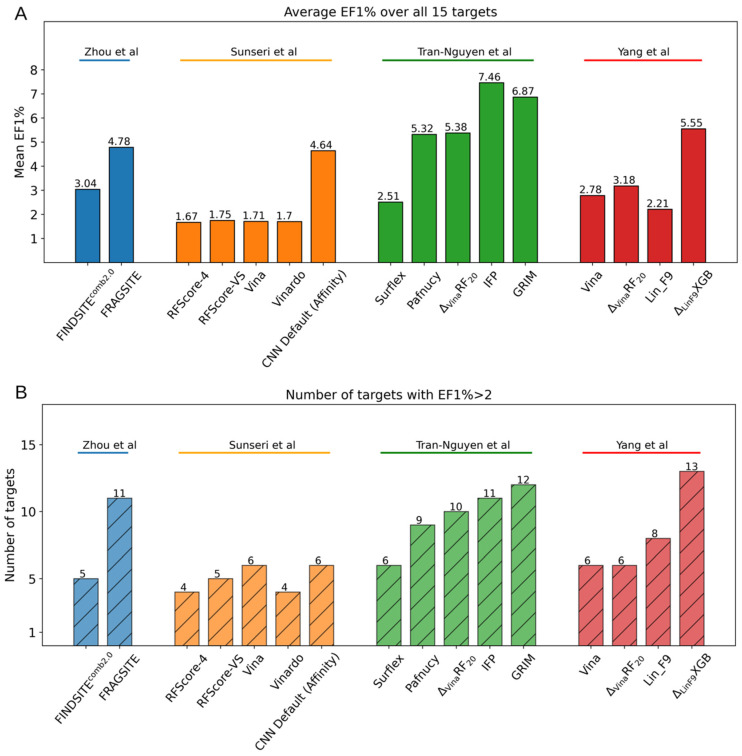
Collected LIT-PCBA benchmark test results from four different groups (Zhou et al. [197], Sunseri et al. [90], Tran-Nguyen et al. [198] and Yang et al. [103]). (**A**) Average enrichment factor at top 1% (mean EF1%) is used to evaluate the early hit enrichment performance. (**B**) Counting number of targets that satisfy the thresholds of EF1% > 2 as a metric to assess the generalizability of the scoring functions on all 15 diverse targets.

Multiple groups have evaluated docking programs and protocols on the LIT-PCBA benchmark (Figure 5) [90,103,197,198]. Tran-Nguyen et al. report the best early enrichment across all 15 targets (average EF1% = 7.46) using the IFP post-docking filtering method. Another post-docking filtering method, Rescoring by Interaction Graph-Matching (GRIM) which compares protein–ligand interaction patterns between a docked and a reference (typically X-ray crystal structure) co-complex structure, also performs similarly well [198]. IFP and GRIM outperform classical and ML scoring function methods in this ranking task but are limited in that they are dependent on the selection of the reference structure and do not predict absolute binding free energy. The Δ_Lin_F9_XGB ML scoring function method lead to the greatest number of targets with EF1% > 2 (13/15 targets) and great average early enrichment (average EF1% = 5.55). Overall, the Δ_Lin_F9_XGB has the best performance among methods that predict binding affinity. In comparison, Zhou et al. and Sunseri et al. report lower early enrichment for their template-based virtual screening methods (FINDSITEcomb2.0 and Fragsite) and their CNN model of GNINA, respectively [90,197]. It should be noted that this comparison of the enrichment results is slightly complicated by the dissimilarity in docking protocols. Sunseri et al. and Yang et al. reported different average EF1% using the Vina docking method likely due to differences in the number of ligand conformers generated, the docking box definition, and the number of PDB templates selected for docking [90,103]. Sunseri et al. used the GNINA software [90], Tran-Nguyen et al. used the Surflex-Dock software [198] and Yang et al. used the Smina software [103].

## 5. Concluding Remarks and Perspectives

The current era is marked by advanced ML techniques, rapid growth of public data and increase in computing power. These developments in computational tools have advanced ML protein–ligand scoring functions for structure-based VS in early-stage drug discovery. Valuable benchmarks and competitions are developed to blindly evaluate these methods. Representative datasets that contain physiochemical data and guide the training of ML methods are proliferating. ML methods have taken advantage of the improvements in computing power and increase in datasets to outperform classical scoring functions. State of the art deep learning architectures applied in other fields are being successfully applied in drug discovery.

Despite these accomplishments, the applications of ML modeling in drug discovery, especially for deep learning, are still in the preliminary stage. Deep learning methods are commonly critiqued for being a “black-box”, easily over-trained, and lack interpretability. To fully appreciate the results, it is required that the user understands the advantages and limitations of a particular model architecture to associate the underlying molecular features to the prediction [199]. It would be valuable to incorporate informative terms and confidence indices to foster the user’s trust in the prediction and indicate starting points for improvements. Furthermore, models are at the volition of high quantities of diverse, high-quality, and curated data. Not only does it require immense collaboration to develop these datasets, but also models may not be able to predict novel associations or characteristics that are not represented in the dataset. Therefore, more attention needs to be given in coupling these technological advances with scientific insights.

The evaluation of these methods and integration of these VS methods in a systematic workflow for prospective study is an active field of research. In recent years, some docking programs have been successfully embedded in automated workflows for ultra-large compound library screening [3,200,201]. However, the selection of promising virtual hits (usually less 100 compounds) from many high scoring compounds in the library remains a challenge, since different selection protocols usually lead to different false-positive rates and mixed hit identification results. We anticipate that future work could try to address these practical problems and limitations in prospective VS studies.

Lastly, scoring functions and SBDD protocols can become more practical and informative as techniques improve. The selection of docking structure and binding site should be done in a systematic manner and consider the functional roles of the particular conformation and binding site. Further investigations of specialized scoring functions for other drug technologies, such as PROTACs, macrocycles, covalent inhibitors, antibodies, allosteric inhibitors and drug combinations, are needed. The scope of structure-based docking protocols can be expanded to predict the toxicity and the cellular responses of the compound. It would be valuable to define protocols that would correlate docking of a particular binding site to the perturbation of the molecular pathways or activity. We expect that ML will play a pivotal role in these areas and continue to influence drug discovery research.

## Figures and Tables

**Table 1:** Machine learning scoring functions.

ML Algorithm	Name	Input Features	Dataset	Year
RF	RF-score [99]	Protein–ligand atom-type pair counts in predefined distance cutoff	PDBbind v2007	2010
	SFCscore^RF^ [100]	Descriptors of ligand-dependent, specific interactions, surface area	PDBbind v2007	2013
	Δ_Vina_RF_20_ [101]	Vina empirical terms, surface area terms	PDBbind v2014 CSAR dataset	2017
XGB	Δ_Vina_XGB [102]	Vina empirical terms, surface area terms, ligand stability terms, bridge water terms	PDBbind v2016 CSAR dataset	2019
	Δ_LinF9_XGB [103]	A series of gauss terms characterizing protein–ligand interactions, surface area terms, ligand descriptors, bridge water terms and pocket features	PDBbind CSAR dataset BindingDB	2022
ERT	ET-score [104]	Distance-weighted interatomic contacts between protein and ligand	PDBbind v2016	2021
GBT	AGL-Score [105]	Algebraic graph theory-based features of protein–ligand complex	PDBbind	2019
	ECIF-GBT [106]	Protein–ligand atom-type pair counts considering each atom connectivity	PDBbind v2016	2021
NN	NNScore 1.0 [107]	Descriptors of specific interactions and ligand-dependent	MOAD PDBbind	2010
	NNScore 2.0 [108]	Vina empirical terms, protein–ligand atom-type pair counts in predefined distance cutoff	MOAD PDBbind	2011
CNN	AtomNet [109]	Local structure-based 3D grid from protein–ligand structures	DUD-E	2017
	Pafnucy [110]	Atom property-based 3D grid from protein–ligand structures	PDBbind v2016	2017
	Kdeep [111]	Atom type-based 3D grid from protein–ligand structures	PDBbind v2016	2018
	OnionNet [112]	Rotation-free element-pair specific contacts between protein and ligand atoms in different distance ranges	PDBbind v2016	2019
GNN	PotentialNet [113]	Atom node feature and distance matrix	PDBbind v2007	2018
	graphDelta [114]	Atom node features considering local environment and distance matrix	PDBbind v2018	2020
	SIGN [115]	Distance matrix of atom nodes and angle matrix of bond edges	PDBbind v2016	2021
